# Bayesian Risk Mapping and Model-Based Estimation of *Schistosoma haematobium*–*Schistosoma mansoni* Co-distribution in Côte d′Ivoire

**DOI:** 10.1371/journal.pntd.0003407

**Published:** 2014-12-18

**Authors:** Frédérique Chammartin, Clarisse A. Houngbedji, Eveline Hürlimann, Richard B. Yapi, Kigbafori D. Silué, Gotianwa Soro, Ferdinand N. Kouamé, Eliézer K. N′Goran, Jürg Utzinger, Giovanna Raso, Penelope Vounatsou

**Affiliations:** 1 Department of Epidemiology and Public Health, Swiss Tropical and Public Health Institute, Basel, Switzerland; 2 University of Basel, Basel, Switzerland; 3 Centre Suisse de Recherches Scientifiques en Côte d′Ivoire, Abidjan, Côte d′Ivoire; 4 Unité de Formation et de Recherche des Sciences de la Nature, Université Nangui Abrogua, Abidjan, Côte d′Ivoire; 5 Unité de Formation et de Recherche Biosciences, Université Félix Houphouët-Boigny, Abidjan, Côte d′Ivoire; 6 Programme National de Santé Scolaire et Universitaire, Abidjan, Côte d′Ivoire; University of Queensland, Australia

## Abstract

**Background:**

*Schistosoma haematobium* and *Schistosoma mansoni* are blood flukes that cause urogenital and intestinal schistosomiasis, respectively. In Côte d′Ivoire, both species are endemic and control efforts are being scaled up. Accurate knowledge of the geographical distribution, including delineation of high-risk areas, is a central feature for spatial targeting of interventions. Thus far, model-based predictive risk mapping of schistosomiasis has relied on historical data of separate parasite species.

**Methodology:**

We analyzed data pertaining to *Schistosoma* infection among school-aged children obtained from a national, cross-sectional survey conducted between November 2011 and February 2012. More than 5,000 children in 92 schools across Côte d′Ivoire participated. Bayesian geostatistical multinomial models were developed to assess infection risk, including *S. haematobium*–*S. mansoni* co-infection. The predicted risk of schistosomiasis was utilized to estimate the number of children that need preventive chemotherapy with praziquantel according to World Health Organization guidelines.

**Principal Findings:**

We estimated that 8.9% of school-aged children in Côte d′Ivoire are affected by schistosomiasis; 5.3% with *S. haematobium* and 3.8% with *S. mansoni*. Approximately 2 million annualized praziquantel treatments would be required for preventive chemotherapy at health districts level. The distinct spatial patterns of *S. haematobium* and *S. mansoni* imply that co-infection is of little importance across the country.

**Conclusions/Significance:**

We provide a comprehensive analysis of the spatial distribution of schistosomiasis risk among school-aged children in Côte d′Ivoire and a strong empirical basis for a rational targeting of control interventions.

## Author Summary

Two types of blood-dwelling parasitic worms that cause schistosomiasis (i.e., *Schistosoma haematobium* and *Schistosoma mansoni*) are endemic in Côte d′Ivoire, West Africa. Reliable information on their geographical distribution is needed to plan and guide the national control program. Recently, control efforts have been intensified. There is a need to update risk maps that, historically, have been based on data specific to each type of parasite. In late 2011 and early 2012, we conducted a cross-sectional survey in 92 schools all over Côte d′Ivoire. We used Bayesian geostatistical multinomial models to estimate the risk for each infection, as well as co-infection. We estimated that slightly less than 10% of school-aged children are affected by schistosomiasis (5.3% with *S. haematobium* and 3.8% with *S. mansoni*). To control schistosomiasis with the deworming drug praziquantel, approximately 2 million treatments would be necessary each year. The distinct spatial patterns of *S. haematobium* and *S. mansoni* imply that co-infection with these two types of parasitic worms is rare across the country. Our results provide a detailed analysis of the spatial distribution of schistosomiasis risk among school-aged children in Côte d′Ivoire, which will inform the national control program for targeted interventions.

## Introduction

The fight against schistosomiasis has been stepped up with global awareness of the burden inflicted upon people who mainly live in rural settings of tropical and sub-tropical countries. Control measures aim to prevent and reduce morbidity due to chronic infection. Whenever resources allow, integrated approaches are advocated that combine preventive chemotherapy targeting school-aged children and other at-risk groups with information, education, and communication (IEC), improvement of sanitation, access to clean water, and focal control of intermediate host snails [1]–[3]. In some countries, long-term concerted efforts successfully controlled morbidity or even achieved interruption of transmission and local elimination [4], [5]. However, the World Health Organization (WHO) minimum goal to regularly administer the antischistosomal drug praziquantel to at least 75% of school-aged children at risk of morbidity is far from being reached (i.e., in 2012, coverage in Africa was only 13.6%) [6]. Schistosomiasis therefore still remains a major public health concern with a conservative 2010 burden estimated at 3.3 million disability-adjusted life years (DALYs) [7].

In Côte d′Ivoire, urogenital and intestinal schistosomiasis are both endemic, caused by chronic infection with *Schistosoma haematobium* and *Schistosoma mansoni*, respectively. Efforts to establish a national schistosomiasis control program date back to the mid-1990s. However, due to the lack of political will and financial resources, and a decade-long socio-political crisis, the program never really took off [8],[9]. In 2010, the “Integrated control of schistosomiasis in sub-Saharan Africa” (ICOSA) project had identified Côte d′Ivoire as a country where preventive chemotherapy is urgently required and should follow WHO guidelines (http://www3.imperial.ac.uk/schisto/wherewework/dfid).

Empirical estimates of the infection risk at the administrative unit where interventions are to be implemented (e.g., health district) are necessary for efficient, cost-effective and sustainable targeting of control measures [10]–[12]. Hierarchical Bayesian geostatistical models provide a robust methodology to establish the statistical relationship between environmental/socioeconomic predictors and the observed risk, while taking into account the spatial dependence inherent to the data. In more detail, it is assumed that the infection risk is driven by a latent spatial Gaussian process, where effects not fully explained by the covariates are captured by a spatial structure in the hierarchy. These models are used in a second step to predict the risk, including uncertainty, at high spatial resolution using Bayesian kriging methods for spatial process interpolation [13].

Model-based estimates reporting about schistosomiasis risk in Côte d′Ivoire come from single species analyses at district [14], [15], national [16], or regional level [17]. Country-wide analyses of schistosomiasis risk are based on historical data that are often heterogeneous [16], [17] and might oversample high endemicity areas as research naturally drives data collection in places where infections are known to be of particular public health concern. Thus, there is a paucity of recent surveys that employed a sampling design that can be utilized for subsequent Bayesian geostatistical analyses of infection risk. Furthermore, the schistosomiasis risk is generally calculated from single species, either using probabilistic laws that assume independence between species [17], [18], or by applying a correction factor allowing for association between species [19],[20]. However, if the data enable the disease outcome to be categorized into different status of infection (i.e., no, mono-, and co-infection), a geostatistical multinomial model can jointly model the different species [21], [22].

In the current study, we assessed co-infection risk with both *S. haematobium* and *S. mansoni* and estimated the risk of schistosomiasis in Côte d′Ivoire by analyzing recent prevalence data obtained from a national cross-sectional survey conducted in 92 schools across the country [23]. We employed a Bayesian geostatistical multinomial model to produce infection risk maps of both *Schistosoma* species, as well as of the overall risk taking into account co-infection. We provide new model-based estimates of the number of infected school-aged children driven by recent data, identify target areas for control measures, and estimate the number of annualized treatments required for deworming the school-aged population.

## Methods

### Ethics Statement

The study received clearance from the ethics committees of Basel, Switzerland (EKBB, reference no. 30/11) and Côte d′Ivoire (CNER, reference no. 09-2011/MSHP/CNER-P), as well as authorization from Ivorian Ministry of Education to conduct the study. Prior to the survey, district health and education authorities, school directors, and teachers were informed about the purpose and procedures of the study. All participants approved verbally their participation to the study and their parents/guardians provided written informed consent. Children infected with *Schistosoma* were treated with a single oral dose of 40 mg/kg praziquantel [1]. In schools where the observed prevalence of schistosomiasis was above 25%, all children were treated with praziquantel regardless of their infection status. Additionally, all children were dewormed with a single dose of 400 mg albendazole [1].

### Study Design and Survey Settings

Details of the study design and survey settings have been described elsewhere [23]. In brief, we designed a national cross-sectional survey, combining parasitological examination, clinical observation, and interviewing school children with a questionnaire. The survey was carried out between November 2011 and February 2012 (dry season), just after the country regained political stability after more than 10 years of political unrest [8].

Study site selection followed a lattice plus close pairs design [24]. In short, we considered 124 grid cells of 50×50 km overlaid on a map that divides Côte d′Ivoire into two ecological zones: a southern forest area and a northern savannah zone. Ecological zone delimitation resulted from an unsupervised classification *via* the “iterative self-organizing data analysis technique” (ISODATA) (for more details, see Schur et al. (2011) [17]). We sampled 54 and 34 grid cells in the southern and northern zone, respectively, proportionally to the population density of the latest available census in 1998. We then randomly selected one locality with a public primary school in each selected grid cell. Six additional school localities were chosen within a 5-20 km radius from the already sampled localities. Teachers of the selected schools were asked to systematically select 60 children attending grades 3–4. If this number was not achieved with classes from grades 3–4, the teachers were asked to select additional children from grade 5. This sample size exceeds the WHO-recommended minimum sample size of 50 for collection of baseline information on helminth prevalence and intensity in the school-aged population within large-scale surveys [25].

### Disease Data

Study participants were asked to provide a stool and an urine sample. Duplicate Kato-Katz thick smears were prepared shortly after stool collection and examined within 45 min *in situ* by two experienced technicians, quantifying *S. mansoni* eggs under a microscope, while microhematuria was assessed using urine using reagent strips (Hemastix, Bayer, UK) as a proxy for active *S. haematobium* infection. Re-examination of 10% of the slides was performed by senior technicians for quality control.

### Environmental, Socioeconomic, and Population Data

Table 1 summarizes sources and properties of environmental and socioeconomic data investigated to estimate the risk of schistosomiasis in Côte d′Ivoire. In particular, we used satellite-derived estimates such as day and night land surface temperature (LST day and LST night), normalized difference vegetation index (NDVI), and rainfall estimates. Climatic variation was accounted *via* the coefficient of variation for rainfall (rainfall cv) and the difference between day and night temperature (LST diff). Soil acidity (pH) and soil moisture expressed supplementary soil characteristics, while additional environmental measures included distance to fresh water bodies and altitude. Ecological zone was accounted as a binary covariate. Socioeconomic proxies were considered with the human influence index (HII) and the percentage of household with improved sanitation [26]. The latter was predicted via Bayesian kriging from household survey data collected by the MEASURE Demographic and Health Survey (DHS), the Multiple Indicator Cluster Surveys (MICS), and the World Health Surveys (WHS) programs. Sanitation facilities were classified as improved following criteria of the Joint Monitoring Program for Water Supply and Sanitation of WHO and UNICEF [27]. Predictions were adjusted for urban/rural classification and for a binary temporal covariate (trend) with a cut-off at the year 2000. Model-based predictions (of improved sanitation) with and without the temporal trend revealed that the trend term was not important and therefore it was not considered in the predictive model of sanitation. School locations were then overlaid to the resulting kriged surfaces to obtain percentage of household with improved sanitation at survey location. The number of school-aged children (age range 5–15 years) was calculated from the Afripop population density database for the year 2010 and used to estimate the population-adjusted risk and calculate annualized praziquantel treatment needs. In the absence of recent census data (the last census had been done in 1998), we considered the Afripop data as the most accurate estimation of the current population.

**Table 1:** Data sources and properties of the variables used to estimate the schistosomiasis risk in Côte d′Ivoire in late 2011/early 2012.

Data type	Source	Temporal resolution	Temporal coverage	Spatial resolution
Day land surface temperature (LST)	MODIS/Terra^1^	8-days	2011	1 km
Night land surface temperature (LST)	MODIS/Terra^1^	8-days	2011	1 km
Normalized difference vegetation index	MODIS/Terra^1^	16-days	2011	1 km
Rainfall	ADDS^2^	10-days	2011	8 km
Altitude	DEM^3^	-	-	1 km
Freshwater bodies	HealthMapper^4^	-	-	-
Soil moisture	WISE3^5^	-	-	10 km
Soil acidity (pH)	WISE3^5^	-	-	10 km
Human influence index (HII)	LTW^6^	-	2005	1 km
Rainfall coefficient of variation (cv)	Derived from rainfall	10-days	2011	1 km
	(standard deviation/mean)			
LST difference	Derived from LST	8-days	2011	1 km
	(day LST - night LST)			
Ecological zone	ISODATA^7^	-	2000–2008	1 km
Improved sanitation	Bayesian kriging of DHS^8^, MICS^9^,	-	1994–2011	1 km
	and WHS^10^ sanitation data			
	with urban/rural^11^ as covariate			
School-aged population (5–15 years old)	Afripop^12^	-	2010	1 km

1 Moderate Resolution Imaging Spectroradiometer (MODIS). Available at: https://lpdaac.usgs.gov/(accessed: 1 October 2012).

2 Africa Data Dissemination Service (ADDS). Available at: http://earlywarning.usgs.gov/adds/(accessed: 1 October 2012).

3 Digital Elevation Model (DEM). Available at: http://eros.usgs.gov/(accessed: 1 October 2012).

4 HealthMapper database. Available at: http://gis.emro.who.int/PublicHealthMappingGIS/HealthMapper.aspx

(accessed: 1 October 2012).

5 ISRIC-WISE database (WISE3). Available at: http://www.isric.org/(accessed: 1 October 2012).

6 Last of the Wild Project version 2, 2005 (LWP-2): Global Human Influence Index (HII) dataset (geographic)

Wildlife Conservation Society International Earth (WCS) and Center for International Earth Science Information Network (CIESIN). Available at: http://sedac.ciesin.columbia.edu/data/set/wildareas-v2-human-influence-index-geographic (accessed: 1 October 2012).

7 Calculated with the Iterative Self-Organizing Data Analysis Technique (see [17]).

8 Demographic and Health Surveys. Available at: http://www.measuredhs.com (accessed: 1 October 2012).

9 Multiple Indicator Cluster Surveys. Available at: http://www.childinfo.org/mics.html (accessed: 1 October 2012).

10 World Health Surveys. Available at: http://www.who.int/healthinfo/survey/en/index.html (accessed: 1 October 2012).

11 Gridded Population of the World version 3. Available at: http://sedac.ciesin.org/gpw/(accessed: 1 October 2012).

12 AfriPop version 2.0. Available upon request at: http://www.afripop.org (accessed: 1 October 2012).

### Multinomial Geostatistical Model

The risks of mono-infection with *S. mansoni*, mono-infection with *S. haematobium*, co-infection with the two *Schistosoma* species, and no infection were jointly modeled with a Bayesian multinomial regression model. Spatial correlation was accounted into the model through stationary geostatistical random effects that were assumed to follow a multivariate normal distribution with variance-covariance defined as an exponential function of the distances between any pair of locations. The overall risk of schistosomiasis is then derived by adding up the co-infection risk to the two species-specific mono-infection risks. Similarly, species-specific overall risks are calculated by the sum of the related species mono-infection and the co-infection. Detailed model formulation is given in the Supplementary Information appendix (S1 Text).

Bayesian inference of model parameters was performed using Markov chain Monte Carlo (MCMC) simulations in WinBUGS version 14 (Imperial College and Medical Research Council; London, United Kingdom). Models were run with one Gibbs sampler chain for 100,000 iterations and the final 1,000 estimates were used for posterior summaries, validation purposes, and prediction at non-sampled locations. Prediction was carried out at 1×1 km spatial resolution using Bayesian kriging over a grid of more than 350,000 pixels in Fortran 95 (Compaq Visual Fortran Professional version 6.6.0, Compaq Computer Corporation; Houston, United States of America).

### Geostatistical Variable Selection

We performed a geostatistical Gibbs variable selection to identify the most relevant predictors to include in the multinomial geostatistical model [28]. Our variable selection procedure was run with one Gibbs sampler chain for 100,000 iterations. Posterior inclusion probabilities were calculated on the last 10,000 estimates of each indicator defining the presence or absence of the covariate in the model. Predictors with posterior inclusion probability superior to 50% defined the median probability model [29]. Further details on geostatistical variable selection model formulation are provided as Supplementary Information (S2 Text).

### Estimated Annualized Treatment Needs

The number of infected school-aged children was calculated for every km^2^ by multiplying the predicted prevalence with the number of children aged 5–15 years. As the Ivorian health system is organized in a pyramidal basis with health districts at operational level, the total number of infected children was summed up over health districts and divided by the total population of children to estimate school-aged children adjusted risk. WHO advocates to administer preventive chemotherapy to school-aged children once a year in high endemicity areas (prevalence >50%), once every 2 years in moderate endemicity areas (10–50%) and twice during primary schooling age in low endemicity areas [25]. To calculate treatment needs on a yearly basis, we assumed an average of 6 years of primary schooling and targeted different proportions of the school-aged children population according to the endemicity level (i.e., the entire, half or a third of the population in high, moderate and low endemicity settings, respectively) [12].

### Model Validation

The multinomial geostatistical model was fitted on a random training sample of 72 locations (around 80% of the full dataset). Predictive ability was assessed on the remaining test locations (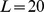) with the mean absolute error (MAE) by averaging the absolute differences between predicted 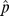 and observed prevalences 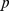, such as 
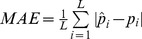
. Predictive uncertainty was measured by summing the standard deviation (SD) of the predictive distributions.

To validate our multinomial geostatistical approach, we developed additional models under different assumptions. We fitted separate binomial models for each parasite species that assume independence between the infections, as well as a non-stationary multinomial model, which considers that spatial correlation is not only a function of the distances between pairs of locations, but also relies on the locations per se. Thus, we modeled the spatial correlation as a weighted average of ecological zone-specific stationary spatial processes [15], [30]. Comparison of the predictive ability of those models with our multinomial model was performed in terms of MAE on the overall schistosomiasis risk.

Our prediction were classified according to WHO thresholds for intervention and we compared the observed prevalence of the surveyed schools with the predicted risk at school location, as well as with the school-aged children adjusted risk at health districts level. Number and percentage of schools overestimated and underestimated were calculated to assess the performance of our model-based estimates.

## Results

### Disease Data

**Figure 1:**
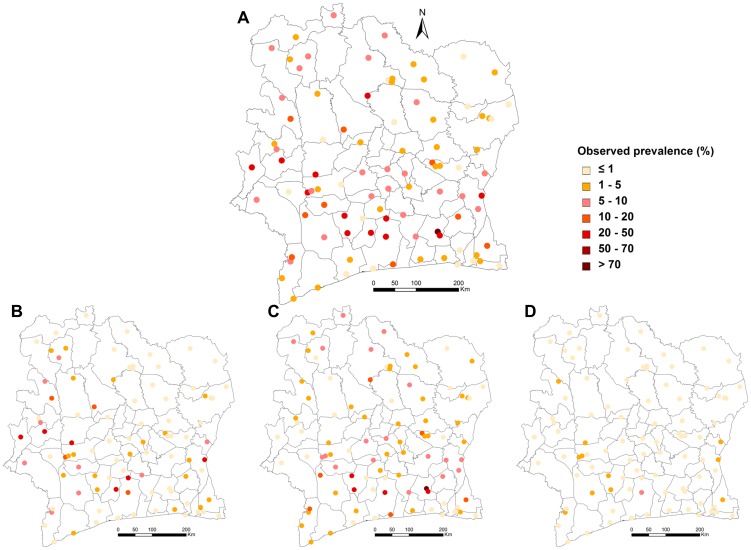
Observed schistosomiasis prevalence in Côte d′Ivoire in late 2011/early 2012. A: Overall schistosomiasis, irrespective of the species; B: overall *S. mansoni*; C: overall *S. haematobium*; and D: co-infection with both species.

Overall, 5,104 children were examined in 92 schools across Côte d′Ivoire. Out of the 94 schools selected, one school refused to participate and another was excluded since teachers reported deworming interventions during the preceding month. Raw parasitological data are provided as Supplementary Information in S1 Table. The mean observed prevalence was 5.7% (standard deviation (SD)  = 11.2%) for *S. haematobium* and 3.6% (SD  = 7.6%) for *S. mansoni* infection. Concomitant infections with both *Schistosoma* species were detected in only 16 children (0.3%, SD  = 0.9%), indicating that *S. haematobium*-*S. mansoni* co-infection is rare in Côte d′Ivoire. The spatial distribution of the overall observed prevalence of infection with any *Schistosoma* species is depicted in Fig. 1, along with the observed distribution of *S. mansoni* and *S. haematobium* single infections, as well as co-infection with both species.

### Geostatistical Variable Selection

Relationships of the 13 potential environmental and socioeconomic predictors with schistosomiasis risk were investigated on the basis of their linear and categorical forms on bivariate non-spatial logistic analyses. Goodness of fit measures showed no benefit to categorize the predictors. Hence, linear predictors were standardized for subsequent analyses. Out of the 13 predictors investigated, LST day was not further considered as the variable was highly correlated to day-night LST difference (correlation coefficient  = 0.94). The median probability model, as well as its posterior probability and posterior inclusions probabilities of the predictors, are presented in Table 2. Ecological zone had a high posterior inclusion probability of 93.6%, highlighting the important difference between the two ecological zones regarding the schistosomiasis risk. The median probability model included ecological zone and rainfall coefficient of variation. Furthermore, it was selected among all possible models with the highest posterior probability. The low posterior probabilities of the models explored by our variable selection (below 3.2%), together with the high inclusion probabilities (above 15%) of all potential predictors, suggest good mixing properties of the MCMC simulations and no clear benefit to choose between the explored predictors.

**Table 2:** Geostatistical variable selection results.

Predictors	Median probability model	Predictor posterior inclusion probability
North ecozone	X	93.6%
Altitude	0	28.9%
Human influence index (HII)	0	15.1%
Soil moisture	0	34.1%
Soil acidity (pH)	0	22.7%
Normalized difference vegetation index	0	15.5%
Night land surface temperature (LST)	0	18.4%
Rainfall	0	39.3%
Rainfall coefficient of variation (cv)	X	60.8%
Day-night difference land surface temperature	0	26.3%
Sanitation index	0	17.4%
Distance to fresh water bodies	0	15.2%
Day land surface temperature	NC	NC
**Model posterior probability**	3.2%	-

X (selected), 0 (not selected), NC (not considered).

Median probability model is presented together with posterior inclusions probability of the predictors and model posterior probability.

### Multinomial Geostatistical Model

A multinomial logistic model, including ecological zone and rainfall coefficient of variation, was fitted to the data. Estimates of the parameters are presented in Table 3, together with predictive ability of the model. Northern savannah ecological zone had a negative effect on the log of the risk of all the multinomial categories *versus* no infection (i.e., *S. mansoni* mono-infection, *S. haematobium* mono-infection, and co-infection with both *Schistosoma* species). Higher rainfall variation had a negative effect on *S. haematobium*, and consequently on co-infection, while its effect was not important regarding *S. mansoni* infection risk. Residual spatial correlation was higher for *S. mansoni* mono-infection (153.2 km) than for co-infection risk (107.6 km), and *S. haematobium* mono-infection (66.4 km).

**Table 3:** Parameter estimates and predictive ability of Bayesian geostatistical multinomial logistic model.

		*S. mansoni*	*S. haematobium*	*S. haematobium-S. mansoni*
		mono-infection	mono-infection	co-infection
**MOR (95% BCI)**	North ecozone	0.32 (0.13; 0.99)*	0.39 (0.17; 0.78)*	0.05 (0.01; 0.40)*
	Rainfall coefficient of variation	0.74 (0.31; 1.47)	0.70 (0.44; 0.99)*	0.37 (0.09; 0.91)*
**Median (95% BCI)**	Range (km)	153.2 (11.7; 473.9)	66.4 (8.4; 264.2)	107.6 (6.1; 655.1)
	_Variance σ_^2^	5.0 (2.8; 10.4)	1.9 (1.2; 3.7)	1.1 (0.3; 4.2)
**Predictive ability (%)**	MAE	5.81	6.06	0.57
	Sum of SD	1.58	1.32	0.07

^*^Significant based on 95% BCI.

Overall schistosomiasis risk: MAE  = 10.0%; sum of SD  = 2.0%.

Multinomial odds ratios (MOR) and median of the spatial parameters estimates are displayed with their 95% Bayesian credible intervals (BCI). Predictive ability is assessed with a model fitted on a subsample of the data (80%) and is reported by mean absolute error (MAE) and sum of the standard deviation (SD) of the predictive distributions.

For comparison, we built two additional models; one without predictors and another one with all predictors (parameter estimates and predictive ability results are given as Supplementary Information; S2 and S3 Tables). The residual spatial correlation was the lowest for each multinomial category in the model with all covariates. This suggests that predictors which have not been selected by the variable selection were able to explain part of the spatial pattern. In addition, our model shows the best predictive ability. While the model including all covariates shows a better MAE regarding *S. mansoni* mono-infection and co-infection with both species, the MAE of the overall schistosomiasis risk is lower. Moreover, our model shows less uncertainty in the predictions as reflected by lower sum of the SD of the posterior predictive distributions at test locations.

Model validation on 20% of observed location also revealed that the multinomial geostatistical model presented in Table 3 predicted better the overall schistosomiasis risk in comparison to a non-stationary multinomial model (MAE: 10.0% *versus* 11.3%), as well as to separated species-specific binomial geostatistical models assuming either independence of the infections (MAE: 10.0% *versus* 11.0%) or dependence accounted through a correction factor [19] estimated from the data (MAE: 10.0% *versus* 11.0%; correction factor = 0.99).

**Figure 2:**
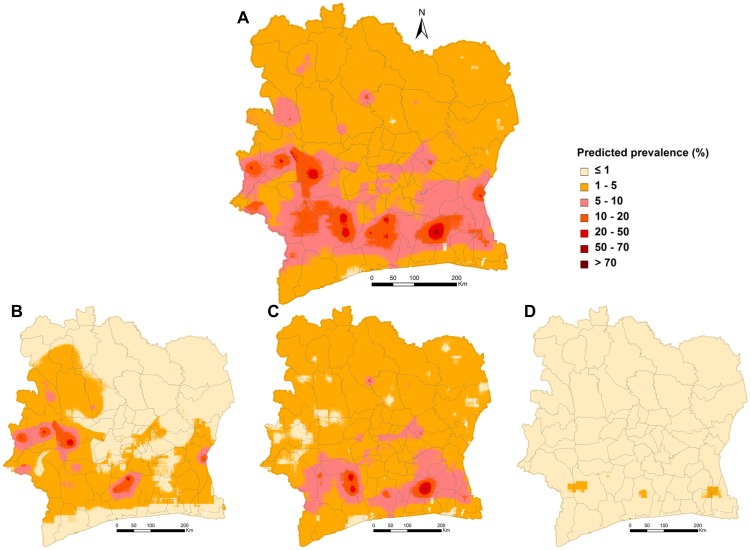
Predicted schistosomiasis risk in Côte d′Ivoire in late 2011/early 2012. A: overall schistosomiasis, irrespective of the species; B: overall *S. mansoni*; C: overall *S. haematobium*; and D: co-infection with both species.

Smooth map of the overall schistosomiasis risk (*S. mansoni* mono-infection, *S. haematobium* mono-infection and *S. mansoni*-*S. haematobium* co-infection) is depicted in Fig. 2A. Maps of the risk of infection of *S. mansoni* and *S. haematobium* (mono- and co-infection) are presented in Fig. 2B and 2C, respectively, while the map of co-infection risk alone is shown in Fig. 2D. We observed that the two species display distinct spatial patterns, which generally do not overlap, and hence, co-infection is low across the country.

### Risk and Estimated Annualized Treatment Need

**Figure 3:**
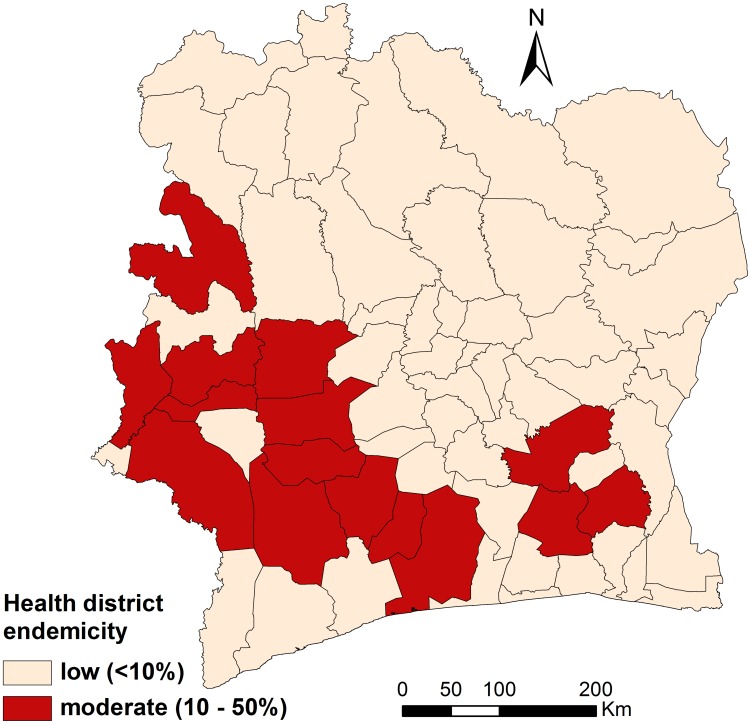
Estimated number of school-aged children at risk of schistosomiasis. Maps derived using WHO guidelines and stratified for health districts for control intervention planning.

In Côte d′Ivoire, we estimated that around 457,062 school-aged children are infected with *Schistosoma*, which correspond to 8.9% of the school-aged population (95% Bayesian credible interval (BCI): 7.5–10.6%; child population aged 5–15 years: 5,135,531). Single species infection risk was estimated at 5.3% (95% BCI: 4.3–6.8%) for *S. haematobium* and 3.8% (95% BCI: 2.9–5.3%) for *S. mansoni*. The children-adjusted risk aggregated at health district level is detailed in the Supporting Information appendix (see S4 Table). The health district of Agboville presents the highest risk estimated to 29.7%. Health districts were classified as low (predicted children-adjusted risk <10%) or moderate (predicted children adjusted risk 10–50%) endemic and the resulting map is presented in Fig. 3. Based on this classification, we calculated that a total of 1,999,629 annualized praziquantel treatments are required for implementation of preventive chemotherapy against schistosomiasis at health districts level in Côte d′Ivoire. High-risk areas extend in the south-western part of the country, as well as in the northern areas of Abidjan. Misclassification of the surveyed schools by the predicted risk at school (pixel) and health districts levels is provided in Table 4. Our estimates of the schistosomiasis risk misclassify 4.3% of the surveyed schools, while our predictions aggregated at health district level incorrectly classify 22.1% of the visited schools.

**Table 4:** Misclassification of the surveyed schools by the predicted risk at school and health districts level.

School estimated schistosomiasis risk	<10%	10–50%	≥50%
Schools underestimated	4 (4.3%)	-	-
Schools overestimated	-	-	-
Schools misclassified	4 (4.3%)	-	-

Health district estimated schistosomiasis risk	<10%	10–50%	≥50%
Schools underestimated	-	6 (6.5%)	1 (1.1%)
Schools overestimated	-	9 (14.5%)	-
Schools misclassified	-	15 (21.0%)	1 (1.1%)

Number and percentage of schools overestimated and underestimated are given according to endemic thresholds defined by WHO for control interventions.

## Discussion

We present a comprehensive analysis of the spatial distribution of schistosomiasis risk among school-aged children in Côte d′Ivoire. Our predictive map of the overall risk of schistosomiasis confirms that the disease is endemic throughout Côte d′Ivoire and provides a strong empirical basis for rational targeting of preventive chemotherapy and other control measures.

To our knowledge, this is the first estimation of the overall schistosomiasis risk that has been based on a joint analysis of the two *Schistosoma* species that occur in Côte d′Ivoire, taking into account co-infection risk. Our analysis presents further insights compared to previous modeling efforts that have been done in Côte d′Ivoire [14]–[17]. In particular, our predictions are based on recent survey data, where survey locations have been sampled in order to provide an optimal spatial distribution for geostatistical modeling. Although “lot quality assurance” sampling [31] has resulted in better predictive performance compared to a geostatistical sampling similar to the one developed in this manuscript, the 92 schools sampled provide a good coverage of the entire surface area of Côte d′Ivoire (322,000 km^2^) and a sound basis to quantify the spatial structure of the risk at national level with limited financial resources.

In this study we put forth maps of co-infection rather than co-endemicity risk. The former gives the probability of simultaneous infections at the individual patient level. The latter gives the probability that both infections are present at a given locality. Co-infection implies co-endemicity but not necessarily the other way round. Thus, spatial patterns of co-endemicity and co-infection are not necessarily the same. In fact the definition of co-endemicity in the literature of spatial epidemiology is confusing. In some instances co-endemicity refers to co-infection in others it is calculated as the prevalence of either infection.

We estimated that 8.9% of school-aged children are affected by schistosomiasis in Côte d′Ivoire. This estimate is considerably lower than previously published predictions. For example, Schur et al. (2011) [17] estimated that 41.8% (95% BCI  = 24.4–60.8%) of the population below 20 years of age is infected with schistosomes in Côte d′Ivoire based on an analysis of historical data in West Africa. With regard to *S. mansoni*, our estimate of 3.8% is also several-fold lower than the previously published prevalence of 11.0% (95% BCI: 8.7–13.8%) that has been calculated based on an analysis of historical data at national level [16]. Historical data mainly stem from surveys conducted for other purposes than risk mapping and highly endemic areas were likely oversampled. The current analysis therefore highlights the importance of a rigorous sampling design and mapping activities before launching a national control program. High quality data obtained from surveys well distributed in space are paramount for accurate identification of diseases distribution and efficient use of limited resources for control [22], [31]. Côte d′Ivoire had not yet begun implementation of preventive chemotherapy at the time of our survey, and hence, it is unlikely to attribute our considerably lower infection prevalence due to control interventions. Artemisinin-based combination therapy (ACT) is freely distributed as a key strategy against malaria in Côte d′Ivoire. The partial activity of ACT against schistosomiasis [32] might play a role, which is currently difficult to quantify and would deserve further research.

Our study has several limitations and they are offered for discussion. First, schistosomiasis is known to be focally distributed, governed by the presence of humans, specific intermediate host snails, and human-water contact patterns [33], [34] and the cross-sectional study design of the present study might not capture well this pattern. Aggregating schistosomiasis risk estimates at health district level revealed important misclassification of the schools (22.1%) within the risk thresholds defined by WHO for interventions. Thus, operational and financial advantages that would provide the targeting of interventions at the level of an existing structure, such as the health districts, is challenging due to the focal nature of schistosomiasis. Given the need to better understand the small-scale heterogeneity through additional surveys [35], the western part of Côte d′Ivoire that is a well-known focus of *S. mansoni* [14], [36], [37], has been selected in 2010 for a 5-year randomized intervention study using different treatment schedules against *S. mansoni*, funded through the Schistosomiasis Consortium for Operational Research and Evaluation (SCORE). It will be interesting to analyze the baseline data from the eligibility study that surveyed 263 villages/schools with about 50 children examined for *S. mansoni* in each village/school using duplicate Kato-Katz thick smears. This analysis might fill an important gap of understanding small-scale heterogeneity of *S. mansoni* in this specific region. Second, parasitological analyses were conducted on the targeted population, i.e., school-aged children, using WHO-recommended diagnostic techniques for intervention decisions [25]. Our estimates further refine our prior knowledge of the schistosomiasis situation in Côte d′Ivoire. It should be noted, however that the WHO-recommended diagnostic techniques have limitations. For example, it is widely acknowledged that the Kato-Katz technique and the urine-reagent strip tests lack sensitivity, especially in low endemicity settings [38], while urine-reagent strip tests have additionally low specificity [39], [40]. As a consequence, it is likely that our data underestimate the infection prevalence due to these diagnostic dilemmas [41].

An important objective of our study was to assess the co-infection occurrence among Ivorian school-aged children, given that both *S. haematobium* and *S. mansoni* co-exist in the country. Only 16 of the 5,104 children examined were co-infected, suggesting that co-infection is negligible. This result implies that potential synergistic or antagonistic effects of mixed schistosome species infections on morbidity [42] are of little public health concern in Côte d′Ivoire. The scarcity of co-infection is mainly due to the specific spatial patterns of the two parasitic infections with minimal overlapping of the two species infection risk, as highlighted by the predicted maps, stratified by species. Parameter estimates of models including all investigated covariates show that *S. haematobium* and *S. mansoni* infections proliferate under specific climatic conditions. We attribute these different environmental effects to distinct ecological habitats of *Bulinus* and *Biomphalaria*, the intermediate host snails of *S. haematobium* and *S. mansoni*, respectively [43].

Towards the end of 2012, the national schistosomiasis control program, with support of the Schistosomiasis Control Initiative (SCI) has started its activities, emphasizing the treatment of school-aged children in high-risk areas, including additional mapping activities launched in December 2013. The current results, along with additional mapping facilitated by an operational research project in the western part of Côte d′Ivoire (sustaining *S. mansoni* control, financially supported by the Schistosomiasis Consortium for Operational Research and Evaluation) and fine-grained national mapping funded through the SCI, have greatly influenced the roll out of the national schistosomiasis control program. Thus, we believe that with the breadth of recent activities in collecting up-to-date schistosomiasis data and the developed infection risk models for Côte d'Ivoire, great support can be provided to the Ivorian schistosomiasis control program in their fight against schistosomiasis. Additional concerted efforts will be required to analyze all the data in a timely manner and discuss the findings with the national schistosomiasis control program manager to guide and spatially target control interventions.

## Supporting Information

S1 Text Multinomial geostatistical model.
(DOC)

S2 Text Geostatistical variable selection.
(DOC)

S1 Table Parasitological data.
(DOC)

S2 Table Parameter estimates of Bayesian geostatistical multinomial logistic model without covariates.
(DOC)

S3 Table Parameter estimates of Bayesian geostatistical multinomial logistic model including all considered predictors.
(DOC)

S4 Table Overall schistosomiasis risk adjusted for school-aged population (5–15 years old), by health districts.
(DOC)

S1 Checklist STROBE checklist.
(PDF)

S1 Alternative Language Abstract Translation of the abstract into French.
(DOC)
