# Supplementary material for: Bayesian Risk Mapping and Model-Based Estimation of Schistosoma haematobium–Schistosoma mansoni Co-distribution in Côte d′Ivoire
Source: PLoS Negl Trop Dis. 2014 Dec 18;8(12):e3407. doi: 10.1371/journal.pntd.0003407 (PMC4270510; doi:10.1371/journal.pntd.0003407)
Supplement: S1 Text — Multinomial geostatistical model. (DOC) [file pntd.0003407.s001.doc]

**Text S1: Multinomial Geostatistical Model**

We define $Y_{jk}$ as the number of infected, $n_{j}$ as the number of screened, and $p_{jk}$ the probability of mono-infection with *S. mansoni* ($k=1$), mono-infection with *S. haematobium* ($k=2$), co-infection with the two *Schistosoma* species ($k=3$), and no infection ($k=4$) at observed location $j$, $j=1, ..., 92$. The sum of mono-infections and co-infection ($\sum_{k=1}^{3} p_{jk}$) represents the overall schistosomaisis risk. Assuming that $Y_{jk}$ is multinomially distributed, $Y_{jk}\sim MN(n_{j}, p_{jk})$, we modeled the log odds of the infection in each multinomial category $l$, $l=1, ..., 3$, *versus* the baseline category (no infection) as a linear function of covariates $X_{j}$ and latent spatially structured Gaussian process $\phi_{jl}$, such as: $\log(p_{jl}/p_{j4})=X_{j}^{T}\underline{\beta}_{l}+\phi_{jl}$, where $p_{jl}/p_{j4}$ is the risk ratio between the infection status and no infection and $\underline{\beta}_{l}$ is the vector of regression coefficients for each multinomial category $l$. The spatially structured random effects $\underline{\phi}_{l}$’s are modeled as follows: $\underline{\phi}_{l}\sim MVN(\underline{0}, \Sigma_{l})$, with variance-covariance matrix $\Sigma_{l}$. Spatial dependency is introduced *via* an exponential correlation function of Euclidian distances between observed locations, i.e., $\Sigma_{rsl}=\sigma_{l}^{2}\exp(-\rho_{l}dist_{rs})$, where $\sigma_{l}^{2}$ is the geographical variability, $\rho_{l}$ control the rate of the spatial decay, and ($r, s$) is a pair of locations. The range indicates the minimum distance at which spatial correlation between locations is less than 5% and is equal to $-\log(0.05)/\rho_{l}$.

Inference was drawn within a Bayesian framework with non-informative prior specification for model parameters. In particular, vague normal distribution was chosen for the regression coefficients, $\underline{\beta}\sim N(\underline{0}, 100)$, inverse gamma distribution $\sigma^{2}\sim IG(2.01, 1.01)$ were adopted for the spatial variance $\sigma^{2}$, and gamma distribution for the spatial decay $\rho$, $\rho\sim G(0.01, 0.01)$.
