# Supplementary material for: Bayesian Risk Mapping and Model-Based Estimation of Schistosoma haematobium–Schistosoma mansoni Co-distribution in Côte d′Ivoire
Source: PLoS Negl Trop Dis. 2014 Dec 18;8(12):e3407. doi: 10.1371/journal.pntd.0003407 (PMC4270510; doi:10.1371/journal.pntd.0003407)
Supplement: S2 Text — Geostatistical variable selection. (DOC) [file pntd.0003407.s002.doc]

**Text S2: Geostatistical Variable Selection**

For each potential covariates $m$, $m=1, ..., 12$, we introduced a binary indicator $I_{m}$ suggesting the presence or absence of the *m*^th^ variables in the model, and a coefficient $\alpha_{ml}$ indicating the size effect of the *m*^th^ predictors with respect to the *l*^th^ multinomial category. Each regression coefficient $\beta_{ml}$ was seen as the product of the indicator $I_{m}$ and its effect$\alpha_{ml}$, such that $\beta_{ml}=I_{m}\alpha_{ml}$. We assumed *a priori* dependence of $I_{m}$ and $\alpha_{ml}$, i.e., $P(\alpha_{ml, } I_{m})=P(\left.\alpha_{ml }\right|I_{m})P(I_{m})$ and assigned to $I_{m}$ a Bernoulli distribution, such as $I_{m}\sim Be(\pi_{m})$ with hyper-parameter $\pi_{m}$ having a non-informative Beta distribution; $\pi_{m}\sim Beta(1, 1)$. A mixture normal distribution was assumed for $\alpha_{ml}$, such as $P(\left.\alpha_{ml }\right|I_{m})\sim I_{m}N(0, \tau_{m}^{2})+(1-I_{m})N(0, \tau_{m}^{2}/\lambda)$, where $\tau_{m}^{2}$ is a variance parameter with a non-informative inverse-gamma prior distribution, $\tau_{m}^{2}\sim IG(5, 25)$ and $\lambda$ is a constant set to 4,000 to shrink the $\alpha_{m}$’s to 0 when the variable $X_{m}$ is excluded from the model.
