# Supplementary material for: Bayesian Risk Mapping and Model-Based Estimation of Schistosoma haematobium–Schistosoma mansoni Co-distribution in Côte d′Ivoire
Source: PLoS Negl Trop Dis. 2014 Dec 18;8(12):e3407. doi: 10.1371/journal.pntd.0003407 (PMC4270510; doi:10.1371/journal.pntd.0003407)
Supplement: S1 Table — Parasitological data. (DOC) [file pntd.0003407.s003.doc]

**Table S1: Parasitological data.** Number of children examined, mono-infected with *S. mansoni*, mono-infected with *S. haematobium*, and co-infected with both species are given by coordinates of survey locations.

| **Coordinates** | | **Children** | **Children mono-** | **Children mono-** | **Children co-infected** |
| --- | --- | --- | --- | --- | --- |
|  |  | **examined** | **infected with** | **infected with** | **(*S. mansoni* and** |
| **Longitude** | **Latitude** |  | ***S. mansoni*** | ***S. haematobium*** | ***S. haematobium*)** |
| -8.130 | 7.262 | 43 | 11 | 0 | 0 |
| -8.030 | 6.578 | 45 | 3 | 0 | 0 |
| -7.711 | 9.785 | 59 | 0 | 4 | 0 |
| -7.655 | 7.760 | 62 | 0 | 1 | 0 |
| -7.608 | 7.649 | 59 | 4 | 0 | 0 |
| -7.500 | 7.408 | 56 | 16 | 0 | 0 |
| -7.495 | 4.921 | 54 | 0 | 2 | 0 |
| -7.492 | 8.734 | 59 | 4 | 0 | 1 |
| -7.353 | 6.745 | 59 | 0 | 0 | 0 |
| -7.324 | 9.546 | 57 | 1 | 1 | 0 |
| -7.315 | 5.272 | 61 | 3 | 2 | 1 |
| -7.313 | 8.289 | 56 | 6 | 1 | 0 |
| -7.285 | 5.364 | 44 | 0 | 6 | 0 |
| -7.250 | 4.487 | 54 | 0 | 1 | 0 |
| -7.202 | 10.014 | 61 | 0 | 3 | 0 |
| -7.123 | 9.357 | 55 | 4 | 0 | 0 |
| -7.003 | 6.252 | 47 | 2 | 6 | 0 |
| -6.952 | 6.731 | 52 | 6 | 4 | 1 |
| -6.941 | 9.609 | 55 | 2 | 3 | 0 |
| -6.870 | 6.763 | 60 | 1 | 3 | 1 |
| -6.786 | 7.111 | 52 | 20 | 1 | 1 |
| -6.745 | 8.818 | 60 | 1 | 0 | 0 |
| -6.736 | 4.721 | 58 | 1 | 1 | 0 |
| -6.736 | 6.799 | 62 | 1 | 2 | 0 |
| -6.627 | 7.848 | 59 | 0 | 0 | 0 |
| -6.602 | 5.784 | 52 | 2 | 1 | 1 |
| -6.601 | 6.469 | 53 | 5 | 3 | 0 |
| -6.401 | 10.477 | 58 | 0 | 3 | 0 |
| -6.244 | 6.898 | 53 | 0 | 0 | 0 |
| -6.189 | 8.062 | 60 | 7 | 1 | 0 |
| -6.172 | 6.240 | 56 | 0 | 22 | 2 |
| -6.136 | 5.008 | 51 | 0 | 0 | 0 |
| -6.110 | 5.871 | 59 | 1 | 27 | 1 |
| -6.058 | 5.309 | 24 | 0 | 1 | 0 |
| -5.837 | 7.790 | 50 | 0 | 1 | 0 |
| -5.795 | 7.157 | 50 | 0 | 3 | 0 |
| -5.787 | 6.185 | 53 | 0 | 0 | 0 |
| -5.685 | 8.777 | 53 | 1 | 10 | 0 |
| -5.666 | 9.577 | 60 | 0 | 4 | 0 |
| -5.614 | 5.879 | 59 | 15 | 0 | 0 |
| -5.604 | 6.652 | 57 | 0 | 3 | 0 |
| -5.567 | 5.123 | 33 | 0 | 0 | 0 |
| -5.421 | 6.378 | 58 | 0 | 2 | 0 |
| -5.323 | 10.050 | 61 | 0 | 4 | 0 |
| -5.298 | 5.797 | 60 | 5 | 9 | 4 |
| -5.291 | 6.181 | 60 | 18 | 0 | 0 |
| -5.266 | 7.223 | 56 | 0 | 3 | 0 |
| -5.251 | 9.108 | 57 | 0 | 0 | 0 |
| -5.246 | 6.812 | 44 | 1 | 2 | 0 |
| -5.171 | 9.065 | 47 | 0 | 1 | 0 |
| -5.159 | 9.137 | 59 | 0 | 1 | 0 |
| -5.148 | 5.213 | 52 | 0 | 6 | 0 |
| -5.121 | 8.219 | 58 | 0 | 0 | 0 |
| -4.949 | 7.613 | 60 | 0 | 3 | 0 |
| -4.932 | 6.258 | 60 | 4 | 1 | 0 |
| -4.900 | 7.131 | 57 | 1 | 2 | 0 |
| -4.809 | 6.854 | 60 | 0 | 1 | 0 |
| -4.715 | 9.442 | 60 | 0 | 3 | 0 |
| -4.667 | 5.800 | 57 | 0 | 5 | 0 |
| -4.648 | 8.639 | 60 | 0 | 5 | 0 |
| -4.568 | 5.320 | 59 | 0 | 1 | 0 |
| -4.494 | 9.120 | 51 | 0 | 1 | 0 |
| -4.316 | 7.363 | 60 | 2 | 10 | 0 |
| -4.297 | 8.271 | 58 | 0 | 2 | 0 |
| -4.249 | 7.284 | 60 | 0 | 1 | 0 |
| -4.222 | 7.692 | 59 | 0 | 1 | 0 |
| -4.202 | 5.902 | 52 | 0 | 41 | 0 |
| -4.158 | 5.824 | 45 | 0 | 16 | 0 |
| -4.158 | 7.294 | 60 | 0 | 1 | 0 |
| -4.156 | 6.751 | 60 | 0 | 5 | 0 |
| -4.065 | 5.346 | 56 | 1 | 0 | 0 |
| -3.779 | 5.203 | 60 | 0 | 0 | 0 |
| -3.772 | 6.222 | 60 | 3 | 5 | 0 |
| -3.760 | 7.258 | 61 | 0 | 0 | 0 |
| -3.738 | 5.509 | 18 | 0 | 0 | 0 |
| -3.683 | 6.653 | 50 | 1 | 4 | 0 |
| -3.658 | 9.603 | 60 | 0 | 0 | 0 |
| -3.571 | 8.553 | 54 | 0 | 0 | 0 |
| -3.452 | 5.288 | 64 | 0 | 0 | 0 |
| -3.381 | 5.406 | 60 | 1 | 0 | 0 |
| -3.371 | 7.637 | 58 | 0 | 1 | 0 |
| -3.350 | 6.382 | 58 | 0 | 3 | 0 |
| -3.306 | 5.280 | 60 | 0 | 1 | 0 |
| -3.274 | 6.664 | 55 | 12 | 2 | 1 |
| -3.260 | 8.347 | 60 | 1 | 2 | 0 |
| -3.209 | 7.135 | 60 | 3 | 0 | 1 |
| -3.157 | 5.604 | 60 | 0 | 8 | 1 |
| -3.112 | 8.299 | 57 | 0 | 1 | 0 |
| -3.068 | 8.285 | 60 | 0 | 0 | 0 |
| -2.997 | 9.270 | 59 | 0 | 2 | 0 |
| -2.899 | 5.182 | 54 | 0 | 0 | 0 |
| -2.705 | 8.593 | 60 | 0 | 0 | 0 |
