# Supplementary material for: Bayesian Risk Mapping and Model-Based Estimation of Schistosoma haematobium–Schistosoma mansoni Co-distribution in Côte d′Ivoire
Source: PLoS Negl Trop Dis. 2014 Dec 18;8(12):e3407. doi: 10.1371/journal.pntd.0003407 (PMC4270510; doi:10.1371/journal.pntd.0003407)
Supplement: S2 Table — Parameter estimates of Bayesian geostatistical multinomial logistic model without covariates. (DOC) [file pntd.0003407.s004.doc]

**Table S2: Parameter estimates of Bayesian geostatistical multinomial logistic model without covariates.**

|  |  | ***S. mansoni*** | ***S. haematobium*** | **Schistosomiasis** |
| --- | --- | --- | --- | --- |
|  |  | **mono-infection** | **mono-infection** | **co-infection** |
| **Median (95% BCI)** | Range (km) | 139.8 (16.5; 372.7) | 82.1 (18.9; 207.9) | 194.7 (10.0; 624.7) |
|  | Variance σ^2^ | 4.1 (2.2; 7.7) | 2.0 (1.3; 3.5) | 1.8 (0.4; 6.4) |
| **Predictive ability (%)** | MAE | 5.85 | 6.96 | 1.43 |
|  | Sum of SD | 7.21 | 8.16 | 7.28 |

Overall schistosomiasis risk: MAE = 41.92%, Sum of SD = 14.73%.

Median of the spatial parameters estimates are displayed with their 95% Bayesian credible intervals (BCI).

Predictive ability is assessed with a model fitted on a subsample of the data (80%) and is reported by mean absolute error (MAE) and sum of the standard deviation (SD) of the predictive distributions.
