# Supplementary material for: Bayesian Risk Mapping and Model-Based Estimation of Schistosoma haematobium–Schistosoma mansoni Co-distribution in Côte d′Ivoire
Source: PLoS Negl Trop Dis. 2014 Dec 18;8(12):e3407. doi: 10.1371/journal.pntd.0003407 (PMC4270510; doi:10.1371/journal.pntd.0003407)
Supplement: S3 Table — Parameter estimates of Bayesian geostatistical multinomial logistic model including all considered predictors. (DOC) [file pntd.0003407.s005.doc]

**Table S3:** Parameter estimates of Bayesian geostatistical multinomial logistic model including all considered predictors.

|  |  | ***S. mansoni*** | ***S. haematobium*** | **Schistosomiasis** |
| --- | --- | --- | --- | --- |
|  |  | **mono-infection** | **mono-infection** | **co-infection** |
| **MOR (95% BCI)** | North ecozone | 0.05 (0.01; 0.35)^*^ | 0.40 (0.22; 1.9) | 0.01 (0.00; 0.14)^*^ |
|  | Altitude | 2.78 (1.21; 9.23)^*^ | 0.66 (0.41; 1.55) | 3.33 (0.37; 56.09) |
|  | Human influence index | 1.42 (0.64; 2.13) | 1.01 (0.65; 1.51) | 0.61 (0.08; 1.97) |
|  | Soil moisture | 2.96 (1.35; 10.71)^*^ | 0.83 (0.48; 1.54) | 2.82 (0.38; 215.94) |
|  | Soil acidity (pH) | 1.66 (0.42; 3.41) | 1.62 (0.89; 3.09) | 0.82 (0.02; 9.75) |
|  | Normalized difference vegetation index | 0.66 (0.35; 1.91) | 1.25 (0.62; 2.36) | 1.05 (0.21; 4.45) |
|  | Night land surface temperature | 0.46 (0.21; 0.79)^*^ | 1.01 (0.55; 1.66) | 0.72 (0.20; 2.63) |
|  | Rainfall | 0.50 (0.22; 1.09) | 1.32 (0.79; 2.12) | 1.40 (0.11; 7.35) |
|  | Rainfall coefficient of variation (cv) | 0.82 (0.51; 1.71) | 0.59 (0.37; 0.90)^*^ | 0.20 (0.03; 1.22) |
|  | Day-night difference land surface temperature | 0.74 (0.27; 2.29) | 0.95 (0.61; 1.70) | 2.01 (0.21; 11.53) |
|  | Sanitation index | 1.61 (0.93; 2.52) | 1.48 (0.83; 2.50) | 2.72 (0.91; 5.67) |
|  | Distance to fresh water bodies | 0.62 (0.26; 1.12) | 0.97 (0.64; 1.32) | 1.07 (0.40; 3.14) |
| **Median (95% BCI)** | Range (km) | 28.9 (5.8; 126.2) | 46.3 (7.0; 224.1) | 79.9 (6.6; 389.0) |
|  | Variance σ^2^ | 5.1 (2.8; 9.2) | 2.2 (1.3; 4.1) | 2.9 (0.4; 42.9) |
| **Predictive ability (%)** | MAE | 5.71 | 6.28 | 0.54 |
|  | Sum of SD | 2.75 | 1.78 | 0.57 |

* Significant based on 95% BCI.

Overall schistosomiasis risk: MAE = 10.67%, Sum of SD = 3.35%.

Multinomial odds ratios (MOR) and median of the spatial parameters estimates are displayed with their 95% Bayesian credible intervals (BCI).

Predictive ability is assessed with a model fitted on a subsample of the data (80%) and is reported by mean absolute error (MAE) and sum of the standard deviation (SD) of the predictive distributions.
