# Supplementary material for: Bayesian Risk Mapping and Model-Based Estimation of Schistosoma haematobium–Schistosoma mansoni Co-distribution in Côte d′Ivoire
Source: PLoS Negl Trop Dis. 2014 Dec 18;8(12):e3407. doi: 10.1371/journal.pntd.0003407 (PMC4270510; doi:10.1371/journal.pntd.0003407)
Supplement: S4 Table — Overall schistosomiasis risk adjusted for school-aged population (5–15 years old), by health districts. (DOC) [file pntd.0003407.s006.doc]

**Table S4:** Overall schistosomiasis risk adjusted for school-aged population (5-15 years old), by health districts.

| **Health district** | **Population** | **Adjusted risk (%)** | **Health district** | **Population** | **Adjusted risk (%)** | **Health district** | **Population** | **Adjusted risk (%)** |
| --- | --- | --- | --- | --- | --- | --- | --- | --- |
| Agboville | 104828 | 29.7 (13.9; 40.4) | Duekoue | 48001 | 9.2 (5.4; 13.9) | Koun-Fao | 47540 | 6.0 (2.9; 9.9) |
| Man | 120509 | 19.2 (5.9; 27.7) | Aboisso | 112209 | 9.1 (5.9; 13.1) | Bouaflé | 90364 | 5.7 (3.4; 9.3) |
| Divo | 200712 | 17.1 (10.4; 22.3) | Toulepleu | 12941 | 8.9 (4.5; 18.8) | San-Pedro | 44782 | 5.6 (3.0; 10.1) |
| Danané | 102695 | 16.2 (5.9; 25.0) | Toumodi | 42991 | 8.7 (5.7; 13.2) | Katiola | 47918 | 5.4 (3.4; 8.8) |
| Vavoua | 102100 | 16.0 (7.7; 25.3) | Dabou | 72090 | 8.4 (5.2; 12.5) | Korhogo | 163577 | 5.3 (3.0; 9.1) |
| Gagnoa | 149079 | 15.8 (9.8; 22.3) | Didievi | 22217 | 8.3 (5.0; 12.7) | Jacqueville | 18968 | 5.2 (2.7; 9.6) |
| Adzopé | 75130 | 15.6 (9.7; 22.0) | Sinfra | 63715 | 8.3 (5.5; 13.4) | Bouaké-Sud | 4807 | 5.0 (2.6; 9.5) |
| Issia | 89248 | 14.9 (10.5; 20.1) | M'Bahiakro | 41183 | 8.2 (5.0; 12.4) | Bouaké-Est | 198225 | 4.8 (2.4; 8.3) |
| Lakota | 54978 | 14.7 (8.7; 20.6) | Yamassoukro | 105453 | 8.0 (4.2; 13.2) | Grand-Bassam | 26643 | 4.8 (2.0; 9.8) |
| Soubré | 58814 | 12.7 (9.0; 18.0) | Dimbokro | 38873 | 7.9 (4.2; 12.6) | Sakassou | 26673 | 4.8 (2.8; 8.3) |
| Abengourou | 115722 | 12.6 (9.6; 17.0) | Tiébissou | 25809 | 7.9 (4.7; 12.3) | Minignan | 19745 | 4.6 (2.3; 8.3) |
| Sikensi | 62618 | 12.2 (8.6; 17.8) | Oumé | 69490 | 7.6 (4.8; 11.7) | Ferkessédougou | 87510 | 4.4 (2.4; 7.7) |
| Akoupé | 46219 | 12.1 (7.6; 18.3) | Madinani | 15332 | 7.1 (4.5; 10.9) | Kounahiri | 8932 | 4.4 (2.2; 8.2) |
| Bangolo | 44581 | 12.1 (5.0; 19.8) | Mankono | 50697 | 7.1 (4.6; 11.2) | Bouna | 55176 | 4.2 (2.2; 7.1) |
| Guiglo | 59828 | 11.0 (7.3; 16.4) | Tabou | 13161 | 7.0 (4.7; 10.6) | Dabakala | 34055 | 4.2 (2.5; 7.7) |
| Bongouanou | 114827 | 10.8 (7.3; 15.7) | Daoukro | 53769 | 6.8 (3.8; 11.7) | Abidjan zone | 1048866 | 4.1 (1.2; 8.8) |
| Daloa | 186980 | 10.5 (6.6; 14.9) | Zuénoula | 54582 | 6.6 (4.4; 10.4) | Bondoukou | 145400 | 4.1 (2.1; 7.3) |
| Touba | 37850 | 10.0 (5.9; 15.9) | Sassandra | 10398 | 6.3 (3.8; 10.6) | Tengréla | 21658 | 4.1 (1.7; 8.5) |
| Agnibilekro | 42481 | 9.6 (5.6; 14.3) | Alepe | 56547 | 6.2 (3.4; 10.1) | Nassian | 10708 | 4.0 (1.6; 9.1) |
| Grand-Lahou | 30082 | 9.6 (6.1; 15.9) | Odienné | 33521 | 6.1 (3.7; 10.1) | Bouaké-Ouest | 17957 | 3.9 (2.0; 6.9) |
| Biankouma | 39013 | 9.2 (5.0; 14.7) | Séguéla | 48981 | 6.1 (3.2; 11.2) | Beoumi | 40392 | 3.7 (2.0; 6.6) |
| Bocanda | 39701 | 9.2 (6.2; 13.2) | Boundiali | 56277 | 6.0 (3.4; 10.2) | Adiaké | 49400 | 3.2 (1.3; 6.6) |

Adjusted risks are given with their 95% Bayesian credible intervals (BCI) and are listed in decreasing order of importance.
