# Supplementary material for: Bayesian Risk Mapping and Model-Based Estimation of Schistosoma haematobium–Schistosoma mansoni Co-distribution in Côte d′Ivoire
Source: PLoS Negl Trop Dis. 2014 Dec 18;8(12):e3407. doi: 10.1371/journal.pntd.0003407 (PMC4270510; doi:10.1371/journal.pntd.0003407)
Supplement: S1 Alternative Language Abstract — Translation of the abstract into French. (DOC) [file pntd.0003407.s008.doc]

**Translation of the Abstract into French**

**Résumé**

***Contexte:*** *Schistosoma haematobium* et *Schistosoma mansoni* sont des schistosomes responsables de la bilharziose urogénitale et intestinale. En Côte d'Ivoire, les deux espèces sont endémiques et les mesures de contrôle sont en cours d'extension. Une connaissance approfondie de la répartition géographique des infections et la délimitation des zones à risque sont les éléments centraux du ciblage géographique des interventions. Jusqu’à présent, la cartographie du risque s’est basée sur des modèles statistiques qui s’appuient sur des données historiques spécifiques à chaque espèce.

***Méthodologie:*** Nous avons analysé les données relatives aux infections à *Schistosoma* chez les enfants d'âge scolaire obtenues à partir d'une enquête nationale transversale menée entre novembre 2011 et février 2012 sur plus de 5000 enfants ivoiriens dans 92 écoles. Des modèles multinomiaux géostatistiques Bayésiens ont été développés afin d’évaluer le risque de bilharziose, en tenant compte du risque de co-infection *S. haematobium*-*S. mansoni*. Le risque estimé a été utilisé afin de prédire le nombre d'enfants nécessitant une chimiothérapie préventive par le praziquantel, selon les directives de l’Organisation mondiale de la Santé.

***Principaux résultats:*** Nous avons estimé que 8.9% des enfants d'âge scolaire ivoiriens sont infectés par la bilharziose (5.3% par *S. haematobium* et 3.8% par *S. mansoni*). Environ 2 millions de dose de praziquantel sont nécessaires pour une intervention médicamenteuse préventive au niveau des districts sanitaires. Les distributions spatiales distinctes des deux espèces *S. haematobium* et *S. mansoni* impliquent que la co-infection par les deux types d’espèce est rare en Côte d’Ivoire.

***Conclusions / Importance:*** Nous fournissons une analyse détaillée de la distribution spatiale du risque de bilharziose chez les enfants d'âge scolaire en Côte d'Ivoire et une base empirique solide pour un ciblage rationnelle des mesures d’intervention.
